# Scheduling Closure Periods Is Not an Effective Management Strategy to Reduce Lampenflora in Show Caves

**DOI:** 10.1007/s12371-023-00788-y

**Published:** 2023-01-21

**Authors:** Elena Piano, Giuseppe Nicolosi, Marco Isaia

**Affiliations:** Laboratory of Terrestrial Ecosystems, Department of Life Sciences and System Biology, University of Turin, Via Accademia Albertina 13, 10123 Turin, Italy

**Keywords:** Tourist caves, Tourism sustainability, Phototrophic biofilms, Show cave management, Cave closure

## Abstract

The conversion of wild caves into tourist sites poses serious threats to the conservation of subterranean environments. Among them, the extensive growth of photosynthetic biofilms induced by artificial lighting—the so-called *lampenflora*—is of particular concern for cave managers. The identification of cost-effective management actions controlling the growth of *lampenflora* is therefore required to preserve the environmental and touristic values of show caves. By taking advantage of the closure period imposed to contain the COVID-19 pandemic, we tested whether 6 months of cave closure could be an effective strategy to reduce the concentration of photosynthetic biofilms on speleothems in four geographically close Italian show caves. We compared the concentration of the three main microorganism groups composing *lampenflora*, i.e., cyanobacteria, diatoms, and green algae, measured in September 2020 with values recorded 6 months after the closure, in May 2021. Although slight variations have been observed across the different sampling sessions, we did not detect any significant effect of the closure period on the overall concentration values of *lampenflora*. Also, we recorded no significant differences in *lampenflora* concentration after 4 months of regular tourist use, in September 2021. Our results suggest that management practices based on regulating visits to show caves are not effective strategies to reduce *lampenflora*. Therefore, management practices aiming at a sustainable use of show caves should focus on the active removal of photosynthetic biofilms.

## Introduction

The first record of tourists entering a cave is dated back to 1633 in the Vilenica Cave in Slovenia (Cigna and Forti 2013). Since then, and especially during the early 1980s, a number of caves around the world have been converted into touristic attractions, the so-called “show caves,” where paying visitors experience the cave environment and its wonders via constructed trails, guided tours, artificial lighting systems, and regular opening hours (Cigna 2019). However, given their peculiar environmental conditions—ultra-oligotrophic habitats, lack of light and nutrients, spatial confinement, low climatic fluctuations, and low levels of biodiversity (Culver and Pipan 2019)—caves are extremely susceptible to anthropogenic disturbances imposed by their conversion into tourist attractions (Cigna 2016), jeopardizing not only their ecological but also their touristic values. Tourists in caves alter the natural microclimatic conditions (Addesso et al. 2022a) in terms of relative humidity, temperature of air (e.g., Cowan et al. 2013; Šebela et al. 2015) and water (e.g., Šebela and Turk 2014), and CO_2_ concentration (e.g., Lang et al. 2015; Pla et al. 2016), which may enhance carbonate dissolution and damage speleothems (Fernandes-Cortes et al. 2010). In addition, they bring propagules of external microorganisms inside the cave (Addesso et al. 2022b), such as fungi and bacteria, which may generate outbreaks in the cave air (Martin-Sanchez et al. 2014; Porca et al. 2011), water (Ando and Murakami 2020; Moldovan et al. 2020), soil (Kukla et al. 2018; Mammola et al. 2017), and especially speleothems (Saiz-Jimenez et al. 2012), with potential repercussions on the entire subterranean ecosystem. Also, the installation of artificial lights in show caves is necessary to allow visitors to enjoy the natural wonders in an otherwise completely dark environment, but at the same time, it allows the growth of a photosynthetic community alien to the cave (Mulec 2019). The proliferation of photosynthetic microorganisms represents a severe threat to the natural heritage of show caves because this photobiota induces dramatic physical, chemical, and ecological damages (Baquedano Estevez et al. 2019). More in detail, photosynthetic microorganisms grow as components of complex biofilms, forming thick green, brown, or grayish patinas on cave walls, with consequent aesthetic damage to speleothems (Mulec 2019). Also, phototrophic microorganisms, especially cyanobacteria, produce exopolymeric substances (EPSs), which induce the adsorption of cations and dissolved organic molecules from the mineral surface causing the deterioration of the substrate (Bruno and Valle 2017). This substantial damage has consequent negative repercussions on the economic profits of the local populations, ultimately challenging the general sustainability of show cave tourism (Cigna 2016).

The identification of cost-effective interventions controlling the growth of *lampenflora* is therefore required to preserve the touristic values of show caves (Baquedano Estevez et al. 2019). Among the possible measures, the implementation of the tourist carrying capacity (Chen et al. 2021; Lobo et al. 2013) or the closure of show caves to the public for some periods (Killing-Heinze et al. 2017) has been demonstrated as effective strategies to re-establish the baseline values of the cave microclimate. Their efficacy is strictly related to the fact that microclimate alterations induced by visitors are temporary and largely depend on cave size (Dominguez-Villar et al. 2010). Conversely, studies conducted in the iconic Lascaux cave demonstrated that microbial invasions are extremely hard to control, and the return of the cave to pristine conditions is compromised even after several years of cave closure (Alonso et al. 2018, 2019; Martin-Sanchez et al. 2014; Porca et al. 2011). However, the effectiveness of cave closure as a control measure for the *lampenflora* proliferation has never been tested so far.

To fill this gap, we took advantage of the 6-month lockdown period imposed by the Italian government to control the COVID-19 pandemic. During this time, all cultural and natural heritage sites, including show caves, were closed to the public. We compared the total chlorophyll-a (hereafter chl-a) concentration of the three main groups of photosynthetic microorganisms composing *lampenflora*, i.e., cyanobacteria, diatoms, and green algae, before and after the closure period, as well as after 4 further months of a regular tourist flux, in four geographically close Italian show caves.

## Materials and Methods

### Sampling Design

**Fig. 1:**
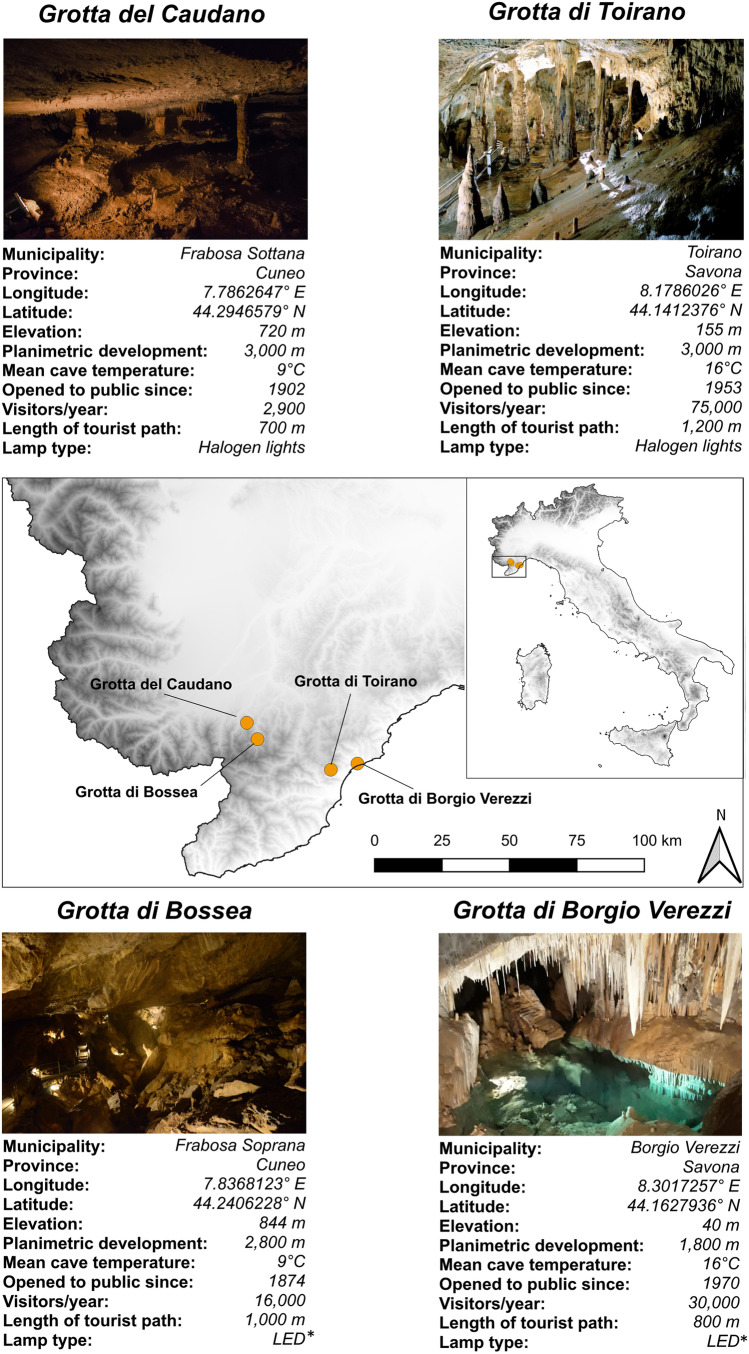
Map of the four examined show caves with information about their characteristics with respect to geography (elevation), morphology (cave planimetric length), physical parameters (mean annual temperature), and tourism (opening year, tourist flux, length of tourist path, and light type). The tourist flux is reported for the year 2019 (photo credits: https://www.grotteturistiche.it). *LED lights have been installed after this study was concluded, while halogen lamps were present at the time of the study

We performed our study in four show caves located at a distance of < 50 km from each other in NW Italy, all opening in limestone rocks, that are Bossea, Caudano, Borgio Verezzi, and Toirano show caves (Fig. 1). Data were collected in three sampling sessions. The first session (hereafter “Control” session) was performed in September 2020, immediately before the cave closure announced by the Italian government to control the COVID-19 pandemic. The second session (hereafter “Closure” session) was performed in May 2021, when all show caves reopened to the public at the end of the national lockdown, for a total of 176 days of closure (closing date = 05/11/2020; opening date = 01/05/2021). We then repeated our measurements 4 months after the cave reopening (hereafter “Opening” session), in September 2021. This third sampling session was included to verify whether the *lampenflora* concentration has recovered to the values observed during the control session after the summer tourist season.

### Data Collection

**Fig. 2:**
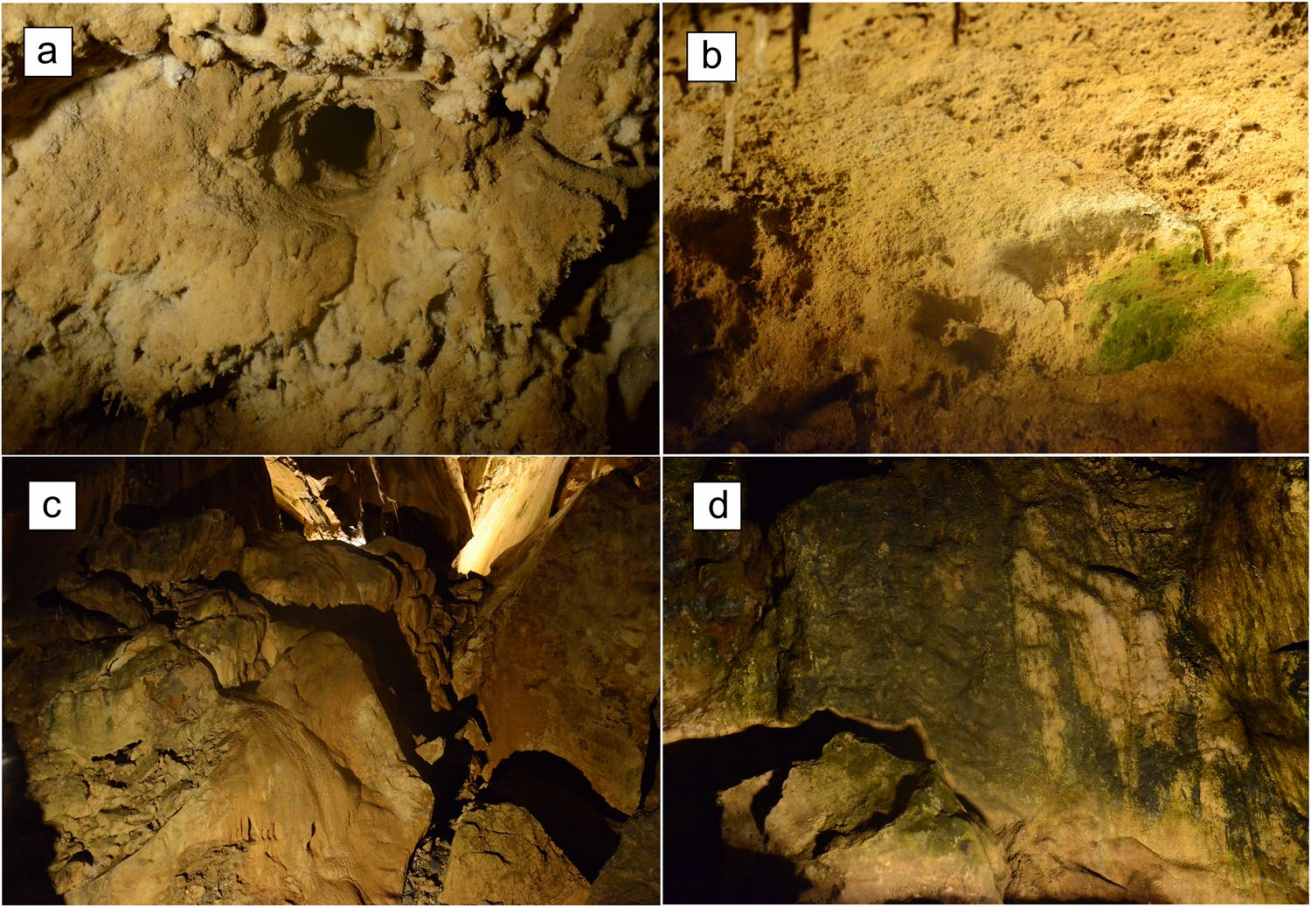
Examples of sampling sites from Grotta del Caudano (**a**, **b**) and Grotta di Bossea (**c**, **d**) monitored during this study (photo credits: Simone Marzocchi)

In each cave, we selected one illuminated speleothem (hereafter “plot”) on average every 50 m from the cave entrance along the touristic path, for a total of 24 plots in Bossea and Caudano, 21 plots in Toirano, and 14 plots in Borgio Verezzi. On each speleothem, we identified one sampling point, consisting in a circle of 20 cm diameter (see Fig. 2 for examples of sampling sites). Sampling points were marked with chalk to be able to perform chl-a measurements exactly on the same site in every sampling session. In each sampling point and in each sampling session, we measured three replicates of *lampenflora* concentration by means of the BenthoTorch®. This instrument, developed by BBE Moldaenke GmbH (Schwentinental, Germany), is a pulse amplitude modulated (PAM) fluorimeter specifically intended to measure the chlorophyll a density (µg chl-a/cm^2^) of the three main photosynthetic organisms, i.e., cyanobacteria, diatoms, and green algae, composing biofilms on hard substrates. The density values are obtained by emitting light pulses at three different wavelengths (470, 525, and 610 nm) and recording the response at 690 nm wavelength. Thanks to an inbuilt algorithm, the instrument calculates an instantaneous and in situ measure of chlorophyll a (chl-a) concentration for each of the three photosynthetic groups.

### Data Analysis

We retained the median value of the three chl-a replicates collected in each plot for each photosynthetic group to be included in the subsequent statistical analyses. The median value was preferred over the mean because it is less influenced by extreme values (Legendre and Legendre 2012). Given that the distribution of the chl-a measures obtained for the three groups was mainly composed by low values with some extremely high values, we used the median values as a dependent variable in the subsequent analyses in order to obtain robust and unbiased results. The total chl-a concentration for each plot was obtained by summing the median chl-a measures obtained for cyanobacteria, diatoms, and green algae.

All statistical analyses were performed with the R software (R Core Team 2021). In a preliminary step, following the approach proposed by Zuur et al. (2009, 2010), we performed data exploration by checking the distribution of dependent variables and identifying possible outliers with the “hist” and “plot” functions, respectively. We considered as dependent variables the total chl-a concentration and the chl-a concentrations of cyanobacteria, diatoms, and green algae, for a total of four dependent variables. The effect of the sampling session was then tested on the dependent variables by means of Generalized Linear Mixed Models (GLMMs), for a total of four different models. Statistical models were performed with the function “glmmTMB” from the *glmmTMB* package (version 1.1.2.3, Brooks et al. 2017). Considering that our dependent variables could not assume negative values, but were highly zero-inflated, we specified a ziGamma distribution, with a log link function, which allowed us to perform zero-inflated gamma models, i.e., ZIGs (Mills 2013). According to the ZIG approach, the algorithm ran a binomial-GLMM, which tests the probability that an outcome is a non-zero value, and a gamma-GLMM, which is appropriate for data with strictly continuous non-zero values. The outcome of the model is composed by two different outputs: (i) the output of the binomial model that explains how the sampling session affects the presence or the absence of the target groups and (ii) the output of the gamma model that reports whether there are differences among sampling sessions in terms of chl-a density values. The presence/absence data are obtained by considering the target group as absent in a sampling site when its chl-a density is equal to 0, while it is considered present when its chl-a density is > 0.

## Results and Discussion

**Fig. 3:**
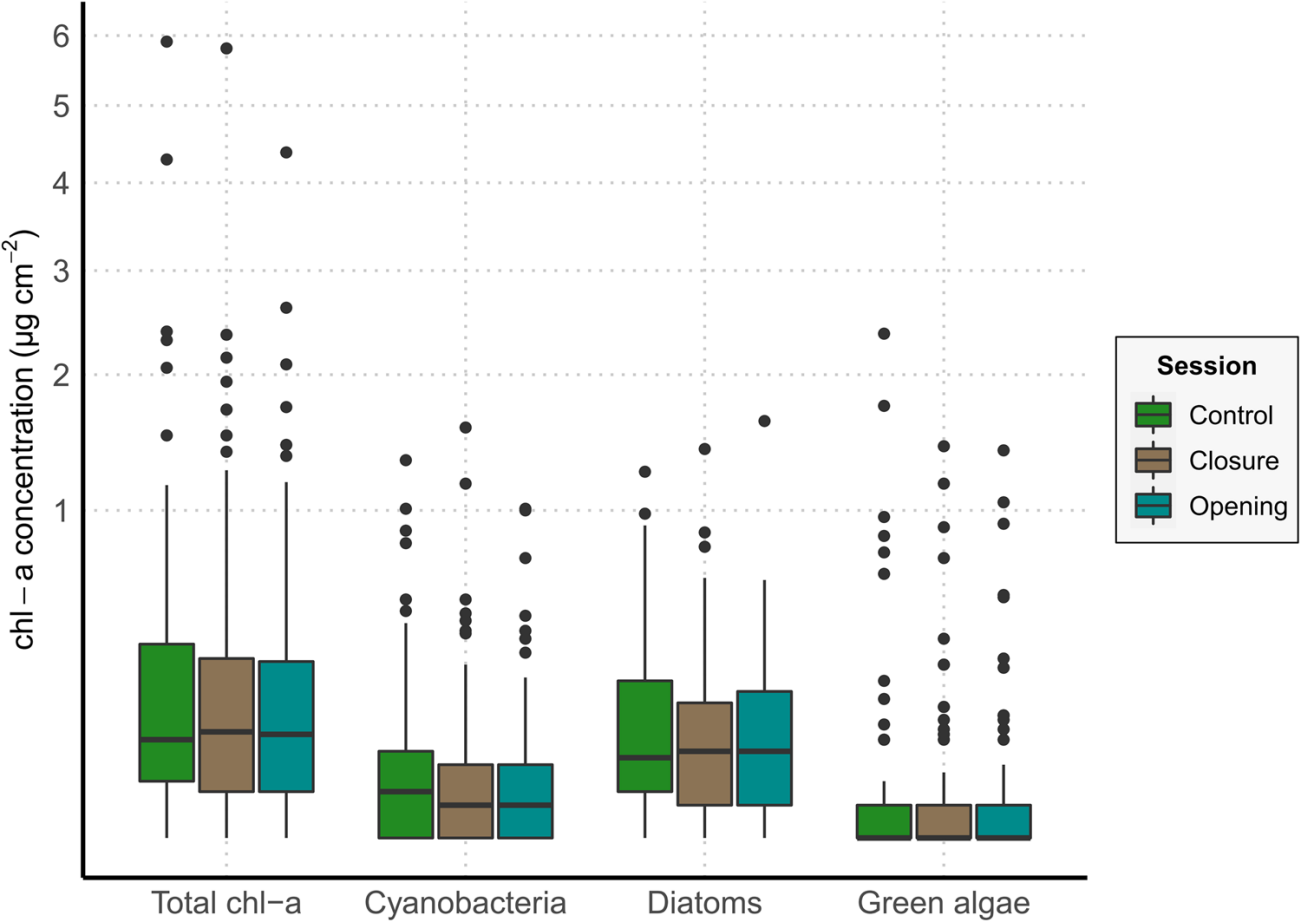
Boxplots representing the concentration values for the total chl-a and the three examined photosynthetic groups in the three sampling sessions. The *y*-axis is square-rooted transformed for a better visualization

The average values of total chl-a concentration and the concentrations of the three examined photosynthetic groups showed some fluctuations across the three sampling sessions (Table 1). Although a slight decrease in the observed values was recorded for all the three groups, as well as for the total chl-a concentration, during the cave closure and after the cave re-opening, we could not detect any significant effect of the sampling session in terms of both chlorophyll-a density (Table 2a) and presence/absence of photosynthetic microorganisms (Table 2b) on any of the examined group, i.e., cyanobacteria, diatoms, and green algae, and on their overall growth, i.e., total chl-a (Fig. 3).

**Table 1:** Summary of total chl-a concentration and chl-a concentration values for each photosynthetic group before and after the cave closure and after 4 months of cave re-opening. Data are expressed as mean and standard deviation (in brackets) of chl-a concentration (µg/cm^2^) and the ranges of the observed values are reported

	*Total chl-a*	*Cyanobacteria*	*Diatoms*	*Green algae*
*Control*				
* Mean (*± *SD)*	0.55 (± 1.35)	0.18 (± 0.68)	0.21 (± 0.45)	0.16 (± 0.64)
* Range*	0.00–9.45	0.00–5.89	0.00–3.56	0.00–4.91
*Closure*				
* Mean (*± *SD)*	0.37 (± 0.79)	0.13 (± 0.45)	0.16 (± 0.32)	0.07 (± 0.24)
* Range*	0.00–5.81	0.00–3.60	0.00–2.21	0.00–1.43
*Re-opening*				
* Mean (*± *SD)*	0.48 (± 1.45)	0.19 (± 1.04)	0.17 (± 0.45)	0.12 (± 0.46)
* Range*	0.00–12.0	0.00–9.36	0.00–2.64	0.00–3.64

**Table 2:** Estimated parameters (*β*-est), standard errors (SE), *z*-values (*z*), and *P*-values (*P*) for the category “Closure” and “Re-opening” for the conditional models (a) obtained for positive values and the zero-inflation models (b) obtained for presence/absence data performed on the total chl-a concentration and the concentration of the three photosynthetic groups. The reference level was the control session. The number of used data (*n*) is reported for each model

	*Variables*	*β-estimate*	*SE*	*z*	*P*
(a) *Conditional models*					
* Total chl-a (n* = *222)*	Closure	− 0.156	0.220	− 0.712	0.477
	Re-opening	− 0.134	0.219	− 0.613	0.540
* Cyanobacteria (n* = *174)*	Closure	− 0.256	0.242	− 1.06	0.291
	Re-opening	− 0.246	0.247	− 1.00	0.319
* Diatoms (n* = *192)*	Closure	− 0.269	0.174	− 1.55	0.122
	Re-opening	0.148	0.174	− 0.834	0.404
* Green algae (n* = *77)*	Closure	− 0.168	0.398	− 0.421	0.674
	Re-opening	− 0.265	0.395	− 0.670	0.503
(b) *Zero-inflation models*					
* Total chl-a (n* = *244)*	Closure	0.387	0.626	0.618	0.537
	Re-opening	0.718	0.610	1.18	0.239
* Cyanobacteria (n* = *243)*	Closure	0.226	0.388	0.581	0.561
	Re-opening	0.226	0.388	0.581	0.561
* Diatoms (n* = *243)*	Closure	0.000	0.482	0.000	1.00
	Re-opening	0.394	0.477	0.711	0.477
* Green algae (n* = *244)*	Closure	0.019	0.355	0.053	0.958
	Re-opening	− 0.184	0.350	− 0.525	0.560

The observed lack of significant difference in the *lampenflora* concentration before (“Control” session) and after the cave closure (“Closure” session) points out that the absence of light experienced during the closure period does not significantly reduce the proliferation of photosynthetic organisms living in subterranean habitats. Our results are corroborated by evidence in literature showing that *lampenflora* can survive at low light intensities or even in the absence of light (Aley 2004; Mulec and Kosi 2009). More in detail, there are studies demonstrating that cyanobacteria, but also fast-growing green algae, such as *Chlorella minutissima*, which are usually the first colonizers in cave biofilms (Mulec et al. 2008; Nikolić et al. 2020), can survive even at extremely low values of light intensity (Czerwik-Marcinkowska et al. 2015; Roldán et al. 2004) in some cases even considerably below the photosynthetic compensation point (Bruno and Valle 2017). Similarly, some aerophilous diatom species can be recorded in dim light or in complete darkness, where the relative humidity of the air is sufficiently high (Asencio and Aboal 2000a, b). Thus, based on our results, we can hypothesize that the extreme conditions of subterranean ecosystems likely exert a strong ecological filter selecting only species adapted to tolerate low light regimes or even light absence. Once these species become established, they can tolerate even abrupt changes in environmental conditions, like the absence of light for long periods.

Our study also pointed out no significant differences between concentration values observed after the cave closure (“Closure” session) and after the summer tourist season (“Opening” session) for all examined groups, even if a slight increase can be observed in the “Opening” session. Although *lampenflora* shows spatial variation in response to local spatial changes in anthropogenic and environmental parameters, such as light intensity, light duration, humidity, and temperature (Borderie et al. 2015; Falasco et al. 2014, 2015; Piano et al. 2015), our results suggest that temporal variations in its concentration are negligible. In other words, photosynthetic biofilms in show caves seem not to be subject to seasonal variations, contrary to what happens in freshwater (Beck et al. 2019; Justus et al. 2021; Piano et al. 2017) and epigean terrestrial ecosystems (Foets et al. 2020) where seasonal changes of ecological drivers, e.g., flow velocity, nutrient availability, and grazer abundance, determine consequent variations in patterns of photosynthetic microbial communities. This lack of seasonality may indicate that the high temporal stability of environmental conditions in subterranean habitats allow the undisturbed growth of photosynthetic organisms to their plateau, without evident changes across the periods of the year.

Although our study is limited to only four show caves, we can reasonably support the hypothesis that scheduling periods of cave closure of less than 6 months does not represent an effective method to reduce the concentration of *lampenflora* in show caves. Considering this, the implementation of adequate management practices is required to control *lampenflora* and contain its repercussions on the subterranean ecosystem. Several studies demonstrated that the modulation of light—that is, the environmental factor easiest to control in show caves—may significantly reduce the growth of photosynthetic microorganisms (Borderie et al. 2014; Bruno and Valle 2017; D’Agostino et al. 2015; Havlena et al. 2021; Mulec 2012; Mulec et al. 2008; Piano et al. 2021; Roldán et al. 2006). However, our results pointed out no significant differences in *lampenflora* concentration when exposed to different lighting regimes—light absence during 6 months of cave closure or regular lighting during the tourist use. Thus, effective mitigation actions aiming at actively removing *lampenflora* are likely required to guarantee the tourist values of show caves. Multiple methods are currently being used, e.g., bleach or hydrogen peroxide solutions (Faimon et al. 2003; Trinh et al. 2018) and UV-C lights (Borderie et al. 2014; Pfendler et al. 2017). Although little is known about their side effects on the subterranean environment, some evidences in literature suggest that they increase proportionally with their efficiency in eradicating *lampenflora* (Meyer et al. 2017). Therefore, their use should be limited to the most compromised speleothems, in combination with an overall modulation of lighting in the entire show cave, e.g., by adopting low-temperature LED (Havlena et al. 2021) or reducing light intensity (Piano et al. 2015) and duration (Piano et al. 2021,2021) and increasing the distance of lamps from surfaces.

## Data Availability

The data that support the findings of this study are available from the corresponding author, upon reasonable request.
